# Opportunities for human factors in machine learning

**DOI:** 10.3389/frai.2023.1130190

**Published:** 2023-04-20

**Authors:** Jessica A. Baweja, Corey K. Fallon, Brett A. Jefferson

**Affiliations:** Pacific Northwest National Laboratory, Richland, WA, United States

**Keywords:** human factors, machine learning, neural networks, data science, artificial intelligence

## Abstract

**Introduction:**

The field of machine learning and its subfield of deep learning have grown rapidly in recent years. With the speed of advancement, it is nearly impossible for data scientists to maintain expert knowledge of cutting-edge techniques. This study applies human factors methods to the field of machine learning to address these difficulties.

**Methods:**

Using semi-structured interviews with data scientists at a National Laboratory, we sought to understand the process used when working with machine learning models, the challenges encountered, and the ways that human factors might contribute to addressing those challenges.

**Results:**

Results of the interviews were analyzed to create a generalization of the process of working with machine learning models. Issues encountered during each process step are described.

**Discussion:**

Recommendations and areas for collaboration between data scientists and human factors experts are provided, with the goal of creating better tools, knowledge, and guidance for machine learning scientists.

## Introduction

Data science has grown at an astounding rate in recent years, especially the subfields of machine learning. With the speed of advancement, there is a need for data scientists to quickly learn new tools, architectures, techniques, and technologies and to learn to address rapidly changing accompanying problems. It is an ongoing challenge for data scientists to remain informed about the latest model architectures and optimization techniques, to develop engineering methods for appropriately handling the volume of available data (or lack thereof), and to use emerging computational and sensor technologies appropriately. With over 250 deep learning articles being released on arXiv alone each month (where peer review is minimal), it is virtually impossible for a single scientist to both be on the leading edge of conducting sound, systematic research and also maintain awareness of the most efficient methods and practices for developing and deploying deep learning solutions. It raises the question of how humans can efficiently conduct meaningful research in this area.

Human factors is defined as the study of human interactions with other elements of a system—and is therefore uniquely situated to evaluate, model, and propose some solutions to the ways that data scientists work and the challenges that they face (De Winter and Hancock, 2021). The understanding provided by a human factors analysis of the machine learning workflow presents an opportunity to develop new tools, best practices, and technologies to improve the process of model development and deployment. Thus, the goal of this effort was to conduct a human factors evaluation of the process data scientists use to work with machine learning models. In doing so, we hope to identify opportunities for human factors researchers to work with data scientists to improve the efficiency and effectiveness of their work.

Data science is defined as the extraction of generalizable knowledge from data, with an emphasis on actionable insights (Dhar, 2013; Igual and Seguí, 2017). As a field of research, data science has exploded in the past three decades, and particularly in the past ten years (Jordan and Mitchell, 2015). The vast increase in the volume of data produced by technology like smartphones, multimodal sensors, and high-performance computers has required new and state-of-the art techniques to process and analyze it, such as machine learning. Deep learning—a subset of machine learning—is a further evolution of machine learning that uses artificial neural networks (ANNs) and representation learning. ANNs, often called just “neural networks,” use a collection of nodes, organized in layers, in which each layer outputs *activations* (a matrix of values) from applying a combination of linear and non-linear functions to the inputs based on the previous layer. The “deep” in “deep learning” refers to the number of layers, where deep learning uses multiple layers of representation to generate an outcome (LeCun et al., 2015).

As mentioned, deep learning relies on representation learning. Also known as feature learning, representation learning generates features or representations of the input data that are needed for detection, classification, or prediction automatically (Jordan and Mitchell, 2015; LeCun et al., 2015). This key distinction means that it is no longer required to specify or engineer the features that might be important to understanding a dataset. Instead, the features are discovered or learned through the data itself. The ability to allow the model to discover relevant features, rather than needing to specify and engineer them, has been critical to the success of deep learning, especially in areas where the data are vast or the important predictive factors are unknown.

The many layers in an ANN used in deep learning help to model more complex data (LeCun et al., 2015). Each layer in the network learns more abstract elements of the data, allowing for more detailed or sophisticated representations. In image classification, for example, the early layers might detect edges, whereas the later layers can learn more sophisticated features, such as objects. However, with the sophistication of deep learning come challenges for interpretability and explainability. Due to the non-linear nature of deep learning and its reliance on representation learning, we lack insight into the reason a model might generate a specific output. That is, it is no longer trivial or straightforward to understand the decisions or recommendations made by the model; instead, significant effort is required to try to deconstruct and explain how the model arrived at a specific output (Goebel et al., 2018). Data scientists can use deep learning to generate powerful outputs, but they are unable to explain it; this has led to growth in the field of explainable AI, which strives to explain how or why a model generated specific output.

Recent work has explored the human factors of data science work (Muller et al., 2019). Muller et al. (2019) interviewed 21 data scientists to understand the ways that human expertise is applied during the process of manipulating and analyzing data. They outlined a variety of ways that data scientists intervene in the data acquisition, cleaning, and feature engineering processes. The authors also described a process over time where data scientists gained knowledge during data cleaning and used it to generate meaningful features and to develop insights from the model. In this perspective, the outcomes of the data science process are a collaborative effort between the data scientist and the machine learning model, rather than an objective output from the model itself. The data scientist intervenes in the model creation process, helping to create and generate meaningful outcomes.

Human factors, which studies the interactions between humans and humans, as well as humans and a system, can likely provide insight into the ways that this process of sensemaking might be improved (De Winter and Hancock, 2021). One aspect of human factors research is task analysis, which seeks to understand the processes involved in the completion of a task and identify the parts of a workflow (e.g., Annett, 2003; Wickens et al., 2004; Stanton et al., 2017). By understanding the step-by-step process by which data scientists work, including classifying the problems and challenges that they face along with how solutions are reached, human factors can help to create a more informed process for data scientists to construct algorithms that work with and complement human information processing capabilities (De Winter and Hancock, 2021). Rather than creating models and machine learning technology that works independently from a human, this collaborative approach strives to create usable and useful data science technologies that work with human users to leverage the unique capabilities of both humans (i.e. adaptability, extrapolation, and more gestalt-centered processing) and machines (speed of processing, accuracy, scale of processing). Perhaps long-term, this interactive construction approach could generate an idealized human-machine team between the machine learning model and the human user (e.g., Groom and Nass, 2007). Human factors helps to inform data science technologies and approaches that optimize the performance of the data scientist and the machine learning model.

Furthermore, the black box nature of deep learning presents an opportunity for human factors in helping to develop explanations for the models. For example, a clear understanding of the audience can help to better explain the technology in a way that is specific to that audience's needs (e.g., Phillips et al., 2020). A clear and comprehensible explanation is useful from a stakeholder or end user perspective, where the explanation provides an understanding for the reasoning or justification for an end result. It is also useful from a data science perspective, where the explanation may help to identify issues in the model that may need to be corrected—as suggested by past work demonstrating on data scientists' reliance on interpretability tools for assessing model performance (Kaur et al., 2020). Improving the practice of machine learning requires generation of more useful explanations for data scientists to understand, interact with, and improve model and algorithmic performance and robustness. This is an area where human factors can readily contribute.

In addition to these more general questions of human interactions with machine learning, there has also been recent discussion of a reproducibility crisis in machine learning and artificial intelligence (Hutson, 2018; Kapoor and Narayanan, 2022). Reproducibility here refers to the ability of the researcher to recreate or reproduce the methods applied, even if the exact results may vary due to some randomness in the modeling approach. There are some challenges in reproducibility in data science due to a lack of emphasis on reproducibility in the field—for example, scientists failing to publish code or include details necessary for replication (Hutson, 2018). Other issues, though, are more related to errors in process that may have resulted from a lack of awareness of important issues in application, such as data leakage, when machine learning methods are applied by domain experts who may lack a complete understanding of the method (Kapoor and Narayanan, 2022). Note that there are some inherent challenges in perfectly reproducing some types of machine learning; some approaches are stochastic, or rely on a random seed, which results in some inherent variability in the results even if the methods themselves are exactly reproduced. There may be other challenges that contribute to a lack of reproducibility of the data science methods even outside of reproducing the exact results. However, further exploration is needed to enumerate the challenges, especially the human challenges, that might contribute to a lack of reproducibility in the field of machine learning.

This study seeks to apply human factors methods to the field of machine learning to understand a general process that data scientists use when developing and working with machine learning models, and specifically ANNs, including the methods and techniques that they apply and the challenges that they encounter. The goal of the study was to understand the ways that data scientists work with ANNs; thus, participants generally used ANNs as a method. Specifically, using semi-structured interviews with data scientists at a National Laboratory, we sought to answer the following questions:

- What is the process or workflow used by data scientists when working with machine learning models?
- What challenges, issues, or problems do data scientists encounter when working with machine learning models?
- How could human factors contribute to addressing issues and challenges working with machine learning models identified by data scientists?

Data scientists were sought who have experience working with machine learning and ANNs, as this subfield of machine learning has specific challenges and opportunities that might be addressed by future human factors research.

## Materials and methods

This project used semi-structured interviews with data scientists who conduct research using machine learning at a National Laboratory. The procedure described in greater detail below is an adaptation of the critical incident technique, and relies on understanding the description of a critical incident (i.e., behaviors that have critical significance on an outcome), the actions taken by the participants, and the changes that they would make in future behavior (Flanagan, 1954). This flexible, qualitative data collection technique can be used to inform a task analysis as well as to understand the challenges, judgments, and decisions that data scientists make during their workflow. Using this technique, data scientists were interviewed and asked to describe a project that used machine learning where their goal was to improve understanding, to simplify, or to improve performance of the model. The goal of the questions during the interview was to understand methods of working with ANNs, including process and challenges.

### Participants

Participants were 11 self-identified data scientists working at a National Laboratory who had experience using ANNs. They were identified using existing researchers' professional networks. Demographics for the sample are shown in Table 1. Participants were, on average, 34.5 years of age (*SD* = 6.7), with nine men and two women. They had an average of 3.7 (SD = 3.6) years of experience as a data scientist with a variety of specialties, ranging from computer vision to physics-informed machine learning. Participants all worked with machine learning, and almost all (*n* = 8) described their specialty as deep learning approaches.

**Table 1:** Participant demographics.

**Age**	**Gender**	**Education**	**Years as data scientist**	**Years at lab**	**Specialty**	**Model type**
29	F	Master's	3	2.5	Adversarial machine learning, record linkage, computer vision	Computer vision
25	M	Bachelor's	3	1.5	Deep learning interpretability, adversarial machine learning	Object detection
47	M	PhD	14	2	Deep learning, Neural data analysis	Neural simulation
23	M	Master's	3	1.5	Deep Learning (Natural language processing [NLP], computer vision); computational statistics	Image classification
31	F	Master's	1.5	1.5	NLP, Question answering, text generation, data cleaning	Natural language processing
34	M	PhD	4	4	Deep Learning	Electrical engineering
29	M	PhD	2	1	Physics-informed Machine Learning	Traffic modeling
24	M	Bachelor's	1.5	1.5	AI/ML, ANNs, Kernel Methods	Computer vision
27	M	Master's	3	3	Deep Learning	Disease modeling
34	M	PhD	5	3	Transformer Models, Explainability, Domain Transfer	Signal detection
32	M	PhD	1	1	Graph Neural Networks, Deep learning	Molecular dynamics

### Procedure

Participants were contacted via email for participation in the study. If they agreed to participate, they were sent a copy of the informed consent document describing the purpose and nature of the study, and once signed, the interview was scheduled. All interviews were conducted via Microsoft Teams and were scheduled for 90 minutes. When possible, two interviewers were present. Due to scheduling constraints, for *n* = 7 interviews, only one interviewer was present; for the remaining interviews (*n* = 4), two interviewers were present. Both interviewers have Ph.D.s in the field of psychology, conduct research that regularly includes human subjects experiments, and have extensive professional experience conducting semi-structured interviews. Finally, both interviewers took notes during the conversation, and in addition, all but one of the participants consented to recording of the interview to facilitate later notetaking.

Interviews were conducted in a semi-structured manner. The interviewer began the discussion by describing the purpose of the study, which was to understand the process by which data scientists work with machine learning models. Participants were asked to think of a specific project and discuss their process. Although participants were not required to discuss an ANN, they were explicitly selected to have experience applying ANNs in their work. Participants were asked to focus on the process; the success in achieving their goals was not important. Instead, participants were asked to think about the research process, techniques they used, and challenges they faced when working with the machine learning model.

After clarifying the purpose of the study and giving the participant an opportunity to ask any questions, the interviewer asked the participants to briefly describe the project that they'd be discussing. The first portion of the interview began with conducting an initial task analysis, asking the participant to describe the overall steps in the process of working with the machine learning model. The interview was conducted using three sweeps, with prompting questions shown in Table 2. These questions were based on the critical incident technique (Flanagan, 1954) with the goal of identifying key decisions during the workflow, ways that anomalies are identified, and cues that participants were using during their process. The goal was to gather a clearer picture of the process used when working with machine learning models, the challenges encountered, and decisions where knowledge or expertise were important. Questions were also asked about the “big picture” or the domain in which the machine learning model would be applied, ways that participants identified anomalies, and how they applied their judgment or knowledge to decisions within the workflow—focusing on the cognitive aspects of a task analysis (Militello and Hutton, 1998). The questions used in the interview were developed based on the critical incident technique and principles of task analysis in order to understand the process of working with machine learning models, the goals of each step, the knowledge and judgment applied, and the challenges encountered by data scientists completing the work.

**Table 2:** Interview questions.

**Sweep 1**	**• Please briefly describe the project that we will be discussing today, breaking your process up into 5 to 7 steps**
Sweep 2	For each step in the timeline... • How long did it take you to do this step? • What analyses are you performing at this step? • Please describe your problem-solving process at this point. • Are you using any tools during this step? If so, which ones?
Sweep 3	• Technology ° Of all these steps in your process where could you use the most assistance? Please describe the kind of help you would be looking for ° If an assistant were to help you, what information would they need to be aware of to help? If you could wave a magic wand to improve any step in your process what are the top things you'd like to change/improve? ° Of all the steps and process you described, what is the most difficult you have to deal with? ° Would you like support from technology during this decision? If so, what kind of support? • Managing Fatigue ° Identify steps in the process that are monotonous. What makes this step monotonous? What do you currently do to manage the monotony? Are there new processes or tools you wish you had to help? • Analysis ° How did you decide how confident you should be in the results of your analysis? If not, were there ways to manage the uncertainty? ° Is there information you wish you had about the model but didn't? ° How did you manage the large number of layers/nodes? Did you have a strategy (e.g., analysis, visualization)? • Noticing/Anomalies ° Are there critical cues you are paying attention to in the model (e.g., particular nodes, levels)? ° How do you know when something is amiss? ° Are you using a strategy or analysis that helped you notice? • What if? ° What if you were under more (less) time pressure when you were working on your model, how would you have adjusted your workflow? ° What if a novice was developing this model? How might they have done things differently? • Prioritization ° How did you decide when to perform the step? • Predictability ° How predictable is this task? • Big picture or domain knowledge ° What is the point in the workflow where it is important for the data to have a big picture understanding of the domain? Why is it important to have a big picture understanding here? ° Are there strategies/analyses you employ to help you?

As the table shows, the first sweep reviewed the general process of the data scientist working with the model, clarifying each of the steps and helping to ensure that the project was a meaningful and useful topic for discussing during the interview. During this sweep, the interviewer essentially worked with the participant to conduct a task analysis, identifying the major steps in the overall task of developing the machine learning model and the goals of each step (Diaper, 2004). In the second sweep, the interviewer asked questions about each step within the process, making sure that the complete sequence of activities was captured. This essentially expanded the task analysis to clarify the subtasks contained within each process step. In the final sweep, questions focused on challenges experienced in each step of the process, areas where assistance might be needed, and where the data scientist might like support with a decision. Retrospective questions were also asked across all steps regarding how the participant might have prioritized tasks differently in hindsight, how they might have approached the problem with more (or less) time, and how domain knowledge or big picture knowledge contributed to their workflow. Not all questions were asked during the interview; questions were asked as applicable based on the nature of the project, the preceding conversation, and the time available.

### Analysis

After all interviews were completed, notes were compiled from both interviewers (as applicable). Recordings were reviewed as needed to add detail to areas where the original notes failed to capture information. Then, all of the responses from participants were compiled into a single document for review. Responses were organized into sections based on the topic of the question. First, results from Sweeps 1 and 2 all focused on the process of working with machine learning models. Responses from all participants were compiled and reviewed. Responses were coded in an inductive manner, with a single author^1^ reviewing and extracting themes from the results. Similarities and differences across the different participants were used to create a generalized description of how data scientists work with machine learning models using a task analytic approach based on the critical incident technique.

Responses to Sweep 3 were reviewed and organized by topic and analyzed using a thematic approach (Braun and Clarke, 2012). Topics were identified in the participant responses and were very evident; participants referred to a specific step when providing a response, and the interviewers asked follow-up questions to ensure that the response was clearly understood. Questions in Sweep 3 focused on a specific step in the process; thus, all responses pertaining to a specific step were compiled for all participants. For example, all responses pertaining to the step of hyperparameter optimization were compiled and reviewed. Short codes were created to capture a participant's response to that topic, and then general summaries or themes were created to describe the pattern of responses across participants.

## Results

Results begin with a description of the kinds of projects that participants discussed during the interviews. Then, we describe each step in the process and associated challenges, followed by some potential recommendations for addressing these challenges.

### Project descriptions

Participants were asked to describe a project where they worked on understanding, simplifying, or evaluating the performance of a machine learning model. All but one of the participants described a project involving use of an ANN. Although data scientists were selected who had experience with and primarily worked with ANNs, we did not require that they discuss an ANN in their interview^2^. Nonetheless, all but one of the participants chose to describe an ANN during the discussion.

In general, the goal of the projects was to optimize model performance for a specific application—that is, the participants were striving to create as accurate of a model as they could within the timeframe of the project. In a single case, the participant was not working with a neural network, but instead the project used a neural simulator and subsequent non-linear machine learning model. Due to concerns about potential sensitivities of some projects conducted at the laboratory, the topics discussed here were limited to publicly available information and focused on commonly used platforms for model development.

The precise output of the projects showed some variability. For most projects, participants were using the outputs of a neural network within an application—that is, the predictions, classifications, etc. themselves were intended for use, at least in demonstration of the capability. In one case, the participant was creating a training algorithm rather than creating a model for application or operationalization. In another case, the neural network itself (rather than the prediction) was the deliverable; thus, although performance optimization was the goal, the intent was to produce a model for later use. This was also true for another project, where the goal was to adapt an existing model to a new software library for future use. In general, however, participants strove to create an accurate model that could be used either in a specific domain application or by future users.

### Process and challenges

**Figure 1:**
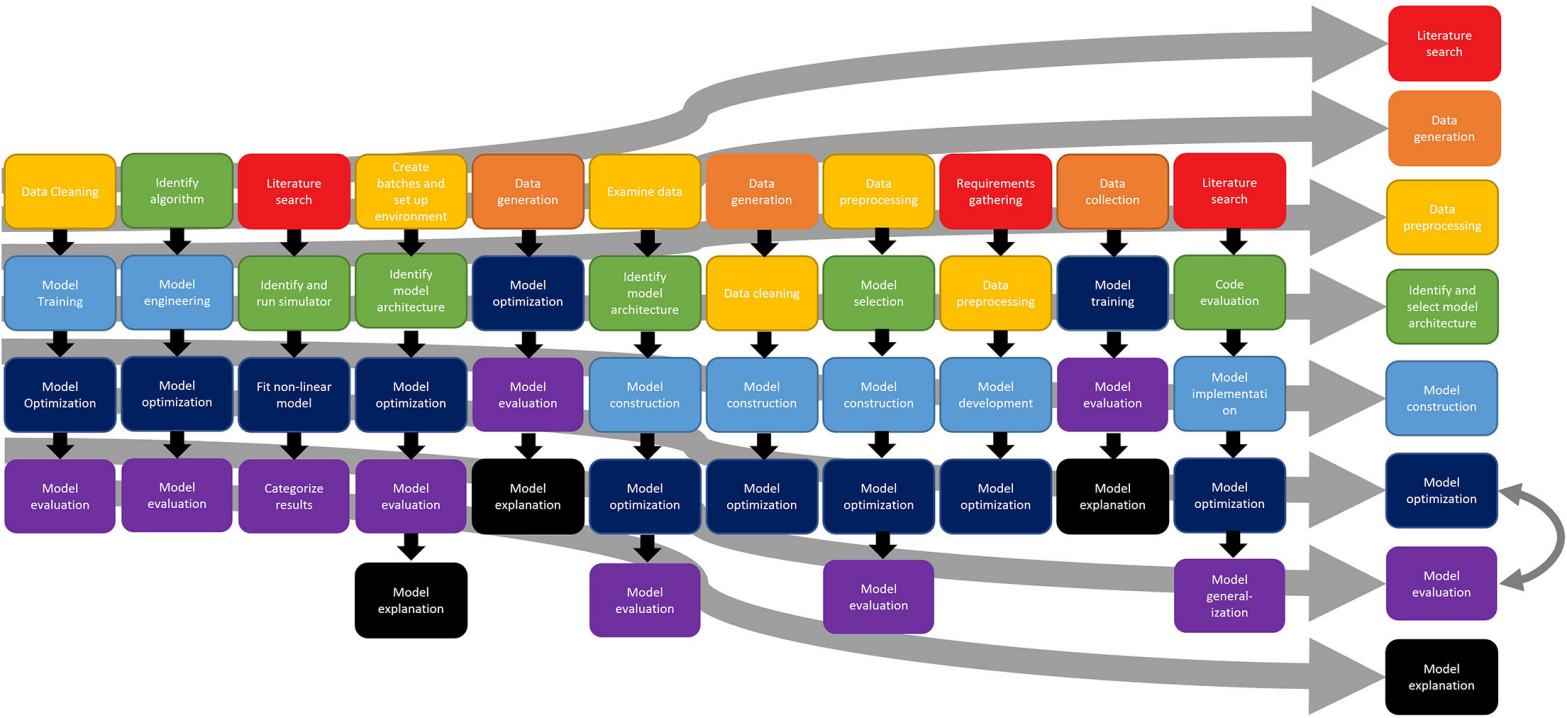
Process of working with machine learning models.

Participants were asked to describe their general process for completing their machine learning project, breaking it down into 5–7 steps. As needed, the interviewer or interviewers repeated past steps back to the participant to assist in breaking down the project into meaningful steps. Participants also often took some time to consider the project overall before beginning their general description. Figure 1 shows this process for all 11 participants, with the right side of the figure displaying a generalized process. This high-level task analysis diagram provides a general depiction of the workflow used by data scientists when working with machine learning models. In creating the generalized process, emphasis was placed on those elements that were most common across the data scientists, assuming that those steps might be the most generalizable; however, that does not necessarily mean that those steps excluded from the generalized process are not important. Rather, it is an indication that those steps might be more idiosyncratic or dependent upon the nature of the application in some way. In addition to describing their process, all participants were also asked about the tools used in each step as well as the time taken to complete each step. Although there were several niche and custom analysis tools (e.g., Julia), almost all participants reported using Python for the implementation of their models, most commonly TensorFlow or PyTorch. The time to complete each step was highly variable depending on the overall timeline of the project. When describing the steps, participants noted that data preprocessing or model engineering (particularly for model implementation on GPUs) were the most time-consuming steps. However, many participants also noted that the model optimization step, and especially hyperparameter tuning, could essentially take as much time as available. That is, they continued this step as long as the schedule allowed. Finally, although displayed in a linear fashion, the steps did not occur in a strictly linear order; many participants discussed moving forward and back through the steps as their knowledge of the dataset, application, and model improved; for example, a particular approach might generate poor results and lead to a different approach. Thus, the figure shows an idealized, high-level process, but not the potential non-linear steps that might occur depending on model performance on other issues. Nonetheless, it serves to illustrate the general steps and process that might occur during the process of working with a machine learning model.

### Literature search

Although many participants described common steps in the process of working with machine learning models, there were a number of differences in the starting point. For example, some participants described the first step as involving some type of planning process, either searching the academic literature to select their model structure or architecture, or gathering requirements, as shown in red in Figure 1. This literature search process (one participant referred to it as requirements gathering, but described a literature search) generally involved reviewing the existing research to understand how other data scientists had approached the same problem in the past, such as understanding model architectures used or hyperparameters selected. This search was generally focused on trying to identify the modeling approach that they thought would be most successful in their application.

### Data generation or collection

One of the common first steps described by participants was the data collection or data generation process, as shown in orange in Figure 1. Although some participants described domain-specific challenges in the data collection or generation process (e.g., experimental challenges), the issues that were described generally arose in the next stage of the process, data preprocessing or data wrangling. The challenges described in the data generation or data collection process were generally specific to the domain application; they may be of use to other data scientists working in that specific field, but they did not reflect generalizable challenges that might be addressed by a process change. Rather, they reflected more specific issues with the specific area of research (e.g., challenges identifying the distance at which a sensor would detect a radio frequency signal). Thus, they are not described in detail here.

### Data preprocessing

Not all of the participants collected the data themselves. Instead, they were given data from another source. These participants began their work on the project in the data preprocessing phase, preparing the data for ingestion into the model, as shown in yellow in Figure 1. Data preprocessing refers to the data cleaning and reformatting necessary to prepare the data for ingestion into the model. This step includes exploring the data format, visualizing the data to identify any out-of-distribution or otherwise problematic values, and feature generation (or identifying those critical parts of the data that the model uses for completing a task) if necessary. Several of the participants expressed challenges or frustration with this step in the process. Scientists working within the domain may have proprietary software or analyze the data in a different format than data scientists, requiring substantial effort to transform the data into the format required for the model. In addition, participants noted that the data preprocessing took more time than expected or more time than they preferred. In general, the sentiment expressed was that this aspect of the data science process is time-consuming, but not rewarding or interesting. Another participant also expressed frustration in identifying differences in different preprocessing techniques—specifically, visualizing the impact of different embedding techniques (i.e., the way that words and phrases are represented mathematically) in a natural language processing task. Preprocessing also created errors in models that were difficult to correct (e.g., introducing invalid values into the data), again, likely due (at least in part) to the difficulty in identifying the impact of different choices on the dataset.

### Model selection

Some participants explicitly described the process of selecting their model. In some cases, this involved determining the type of neural network that they would use (e.g., recursive neural network, convolutional neural network). Other participants described the creation of the model architecture during this step, such as the number of layers that they would use or the number of nodes within each layer. Participants based the decision about which model or architecture to use based on the literature, the examination of their data, or a combination of the two. Although this step is similar to the end point of the literature search, not all participants used the literature search alone to select their model; some participants viewed the data in addition to being informed by the past research in the field.

### Model construction

Almost all participants described model construction or engineering as the next step, as shown in light blue in Figure 1. This step includes the creation of code for implementing the actual model as well as any code or steps needed to create the necessary computing environment.

When discussing the model construction process, a few issues were identified. First, participants discussed challenges in setting up environments. They noted that environments are sometimes not well-documented when reproducing past work. Although the authors may mention the packages used, they may not document versions. Given the pace of deep learning innovation, backwards compatibility with older versions of necessary programming components/ modules can be an issue. The lack of backwards compatibility can make documentation of versions of different packages an important factor during model implementation, and participants reported that this is not done consistently in the literature. Participants also noted that tools designed to facilitate environment setup (e.g., docker) are not consistently used or do not always work as intended. In addition, when attempting to reproduce past research, participants found that the hyperparameters used in the implementation were not always well-documented, making it difficult to obtain the same results as the published study.

Finally, participants also mentioned that, in some cases, implementation of the model on GPUs was challenging, because not all had received training in managing high-performance computers. Many reported learning those skills on the job, making it a time-consuming and challenging part of the project for them.

### Model optimization

Getting models ready for deployment often includes changing the mathematical structure, setting values for model variables (hyperparameters) that govern process variables, and addressing hardware concerns for speed and efficiency. Optimization of the model, hyperparameter tuning, or performance optimization (e.g., computing time efficiency), was described by all participants (as shown in dark blue) as one of the next steps in the process of working with machine learning models. Note that this process is iterative with the model evaluation process; when attempting to optimize their model, participants reviewed the results and adjusted accordingly. Thus, these two steps in particular interacted, as shown by the double-headed arrow in Figure 1.

Participants often mentioned the process of hyperparameter optimization as an area where they could use the most assistance. Hyperparameters within a machine learning model define how the model will learn (i.e., the learning process). If the hyperparameters are not tuned correctly, the model will not minimize the loss function, leading to suboptimal results. Thus, they are important to achieving the best possible performance of the model. Participants described the difficulties in model optimization and discussed some potential solutions to those challenges. Participants expressed a desire for standardized, best practices for hyperparameter tuning. A manual or guide was described that would help individuals know which hyperparameters would be useful in a specific architecture or domain application, or outlining the values that should be explored. Although some tools exist, participants consistently expressed that the tuning of hyperparameters continues to be challenging. Specifically, given the number of possible hyperparameters and ranges, participants reported lacking confidence or knowledge about which values should be tried. In one case, a participant noted that a failure to explore a specific range of values for a hyperparameter resulted in less-than-optimal performance. Because there was no guidance around what values should be tried, and no practical method to explore all of them, it was not clear that additional exploration would have been beneficial. Overall, participants expressed a lack of knowledge or confidence for which values to explore and which were optimal given their application.

Relatedly, participants also highlighted the lack of documentation of hyperparameters as problematic, both for their own work and for others'. Hyperparameter exploration is often a long process of trial and error and documenting the hundreds of combinations is often difficult given the high dimensional space scientists are exploring. First, within their own work, participants expressed challenges documenting the various hyperparameters that they tried within their model. Although some noted that tools are available for tracking hyperparameter exploration, they were not consistently used, suggesting that those tools could be improved. Second, participants noted that articles frequently failed to report the range of hyperparameters that were used within the model optimization process, making it hard to know what values should be used when conducting their own replications or extensions of existing work. Some participants expressed that they were confident in their own results only because they were comparable to the work of others in the same area—that is, they reached similar levels of accuracy as existing work. If no comparable results were available, they were not confident in the results because they felt ill-equipped to determine when the model was reaching the highest level of performance without a baseline for comparison. Finally, participants also noted that the challenges with hyperparameter tuning also create issues with reproducibility of data science work. Because decisions are not necessarily documented consistently, including hyperparameter exploration, the results or findings of a study might not be reproducible.

### Model evaluation

Model evaluation involved evaluating the performance of the model using accuracy metrics. When asked about confidence in the results of their models, participants described a few techniques. First, traditional visualizations and metrics like loss curves and accuracy were mentioned as helping them to assess the quality of their model. Participants reported using the known metrics to assure themselves that the findings they produced were accurate. That is, they evaluated the quality of their results using the accuracy metrics that had been achieved in previous similar research.

In addition to using objective accuracy metrics, participants also discussed exploring the data to help them to assess *why* the model was accurate (i.e., whether it was accurate in meaningful ways). As one example, in an image classification task, the participant described evaluating accurate classifications to ensure that the classes were not created based on some nuisance or noise parameter (e.g., the background of the image, a watermark). Another participant reported testing the limits of a model by running it on a new dataset to assess its robustness. This suggests that the participants were seeking insight into the model in order to determine how it was making decisions. They did so by either testing performance under different conditions, or by attempting to explore the data in each class and develop intuitive explanations for how or why the model reached the results that it did. If they could understand and generate explanations for model performance, they were more confident in their results.

Finally, participants noted that they sought evidence that their findings were consistent with other researchers to instill confidence in their accuracy. Specifically, they reported that they felt more confident in their results when they found additional research or literature that found the same results, or when another researcher attempted the same problem and got comparable findings.

### Model explanation

A small portion of the data scientists in this study included model explanation as the end point of their model. Although not all reported the step of model explanation as part of their process, however, others did describe some portion of explainability when discussing the evaluation of the model. For example, even if not explicitly highlighted as a step in the process, several data scientists mentioned returning to the data to try to develop intuitive explanations as to why images were classified by the model in a specific way. This was generally done as part of the evaluation of the model to assess whether the model was performing well for the right reasons, or to understand how the model performed under different conditions. In the model evaluation process, this information was used to try to assess whether the model should be considered to be performing well (i.e., if it was working as desired or expected); here, the data scientists described trying to understand the reasons for the results provided. Although there were no explicit explanations in this exploration, they nonetheless represent an effort to explore and understand why the deep learning model was producing the results that it was.

## Recommendations and areas for collaboration

Participants identified a number of issues that occurred during the process of working with machine learning models that could be supported by additional tools, technologies, or changes in process. These challenges are summarized in Table 3, along with a representative note from an interview describing that challenge. These notes are not verbatim, but help to provide the character of the comments that led to that specific challenge or theme. The sections below provide some recommendations and areas for collaboration between the fields of machine learning and human factors to help address those challenges.

**Table 3:** Representative notes for each challenge.

**Challenges identified**	**Representative quote**
Involving data scientists in data collection	I always think it's going to be quick. You give me the data, I play around with it, I look at it. Usually you think it would be 10-20% of the project, but it's always longer than that. When they generate the data, it takes them a long time. If they give it to me, and I don't think it's clean enough, I send it back and ask for it in a different format. The format that the [domain scientists] are familiar with I'm not familiar with. Even if they gave that to me, and I look at the data and try to wrangle it a little bit, I realize that there's something wrong with the data—some missing data or something like that. Until I have the clean data, it's usually double what I think the time it would take. That part, if you consider from the moment they run their simulation to the moment I can actually do data analysis, for the project time, maybe I would say it could be like half. The data, getting the data, usually takes longer than I thought it would.
Improved tools for hyperparameter optimization	Data scientists all use different methods to look at hyperparameters. There are still a lot of decisions that are manual, although we're trying to standardize. The differences between data scientists in how they make those decisions do impact the model substantially. For example, [a previous mistake] I made where I didn't test a low enough learning rate, when we changed that, the model changed substantially—it increased 10% in accuracy.
Computing and environment challenges	Setting up environments is monotonous and challenging. Some people are amazing and publish a docker file and these are files that you can run, and they'll set up the entire environment with the exact libraries you need. Those have a tendency to break too, which I'm not sure why or how. When that happens and you have to set up your libraries yourselves...I might have three versions of PyTorch but this one needs another and so I need to download it. That's the most monotonous.
Model explanation for data scientists	During the data cleaning process, we had to resize all the images to be the same size, but they weren't originally. In addition, some of the images were very blurry; others were very clear. When evaluating the model performance, we noticed that the model might then recognize the images that are blurry as being from a specific class. There were other issues like that, where some images had a lot more black, others had a lot more light, etc. You start to realize that there might be some bias because of those features in the model that you don't want it to pay attention to. You need to make sure that, even if the model is performing well, it's performing well because of a reason that you want.
Developing expertise in machine learning	In hindsight, I might have tried some different hyperparameters or data augmentation if someone else had suggested it. Time pressure wasn't a big issue. I have more experience now. At the time, I didn't think to try some things because I didn't have the experience.
Domain knowledge	The project was [the domain expert's] idea. He knows what [domain] analysts do and the difficulties they face, and he knows that language models are quite appealing to them. That's how this project was born and it wouldn't have happened without domain knowledge.

### Involving data scientists in data collection

One of the challenges that data scientists in this study emphasized was the amount of time that they spent preprocessing or “wrangling” the data as described in Section 4.2.3. It was frequently described as being the most time-consuming aspect of the project. Thus, although they were not involved in how the data were collected or stored, they were nonetheless heavily impacted by it. A logical suggestion, then, is to involve data scientists during the data generation (e.g., through simulation) or data collection stage. Involving data scientists consistently during this step of the process could help to reduce the issues caused by the way that data are structured or stored.

This is especially true considering that the results of this study and past work suggest that data scientists are intimately involved in the curation and sensemaking process when data wrangling (e.g., Gitelman and Jackson, 2013; Muller et al., 2019; Sambasivan et al., 2021). As Bowker put it, “Raw data is both an oxymoron and a bad idea; to the contrary, data should be cooked with care” (Bowker, 2008). Collaboration between data scientists and the team collecting the data is valuable because it provides context and a deeper understanding of the data to the data scientist. Knowledge of the data provenance, context around data collection, and awareness of any domain issues can benefit data scientists as they wrangle data, allowing them to generate more meaningful features. From the converse perspective, involving data scientists in the data collection process might also make the later data wrangling process more efficient by giving data scientists some control over how the data are structured or stored in the first place. The process map shown in Figure 1 suggests that data scientists still are not consistently involved in the data collection process. However, including data scientists in the data collection process would likely improve the efficiency of the data wrangling process and the quality of resulting data.

In many cases, data scientists are analyzing data that were collected for another purpose. In this case, there may be little opportunity for the data scientist to be involved in how those data are constructed. In those situations, the results here emphasize the importance of gathering as much information as possible about how the data were produced and their meaning so that they can be used correctly. Ideally, this would involve conversations between the data scientist and the data creators. This is an area where human factors researchers might contribute, by working with data scientists to help create a template or a list of information that might be useful to know when working with a previously created dataset for machine learning purposes. This might be especially useful consider that it may not always be possible to have direct involvement in data creation or structure.

### Improved tools for hyperparameter optimization

Hyperparameter optimization is a known challenge in machine learning (Yang and Shami, 2020; e.g., Cooper et al., 2021). Participants here almost universally highlighted hyperparameter optimization as being a time-consuming, ambiguous, and challenging process, as discussed in Section 4.2.6. Although certainly this is an area where data science is innovating and building tools to try to better identify or document hyperparameters (e.g., HyperTuner; Li et al., 2018), it also represents an area where collaboration between data science and human factors experts might be beneficial. Specifically, additional and more detailed analysis of the hyperparameter tuning process and data scientists' needs could help to inform the tools that would benefit data scientists during this process. This detailed task analysis could also help to identify any best practices or standard operating procedures in the hyperparameter optimization process. In addition, human factors experts can help to evaluate existing tools or libraries to contribute to their enhancement through surveys and user studies. Certainly, the work exploring algorithms and libraries for hyperparameter optimization is a critical piece of addressing this technical challenge; however, the results here suggest that additional work from a human factors perspective to identify the needs for tools and technology in the process would benefit the data science community.

Notably, the challenges with hyperparameter optimization have consequences above and beyond model performance. Difficulties knowing which hyperparameters were explored or selected can also produce challenges with reproducibility of work, which our participants also mentioned. Working with human factors experts to identify systematic ways to explore, select, and document hyperparameters therefore has potential benefits not only for the performance of machine learning models, but also for the reproducibility of work in the field.

### Computing and environment-related challenges

When setting up to run a model, data scientists expressed a few challenges. First, many of them lacked training in how to use graphics processing units (GPUs), as discussed in Section 4.2.5. As a result, many reported needing to learn on the job. Although this is not necessarily problematic, it does suggest that one way to improve efficiency in the process of building complex machine learning models is to incorporate training on high-performance computing resources into professional development. It could be integrated into coursework in data science or provided by employers for early career resources. Alternatively, for organizations that work with high-performance computing resources, providing ongoing training and resources about how to use those resources could help to train data scientists as they learn this new skill.

In addition, data scientists in this study also noted that they sometimes had difficulty in setting up environments to properly run models to replicate what they found in the literature. These difficulties generally occurred because either the environments were not thoroughly documented or because newer or alternative versions lack backwards compatibility. Obviously, the issues here are complex: there are data science challenges associated with reproduction of environments (e.g., through tools like Docker) and with version compatibility. However, there is also a potential opportunity for collaboration between human factors researchers and data scientists to develop additional tools or resources that support documentation of environments or versions. Given the complexity of this issue, it is likely that both approaches will be necessary to address this challenge. Similar to the challenges with hyperparameter optimization, the challenges with backwards compatibility or computing environments also have implications for reproducibility of machine learning research; addressing these issues could help to support more reproducible work in the field.

### Model explanation for data scientists

The focus on explainable artificial intelligence (XAI) has increased as machine learning has been applied in higher-stakes domains (e.g., medicine). In these domains, there is a need to understand not only how accurate the model is, but also how or why it came to that conclusion. This literature often emphasizes the needs of decision makers or end users when providing an explanation (e.g., Hoffman et al., 2018). However, the results here also indicate that data scientists themselves can be the audience for an explanation insofar as they seek understanding of how or why their model performed a specific way to improve that performance. In addition, past work has also recognized that explanations are not one-size-fits-all (e.g., Hoffman et al., 2018; Phillips et al., 2020), but must be tailored to audience needs in order to be meaningful. This suggests that explanations of machine learning models may need to be tailored to data scientists as an audience in addition to focusing on stakeholders or end users. Data scientists may require different types of information about the model for those explanations to be meaningful.

The results here provide some guidance around what meaningful explanations for data scientists might be. The data scientists interviewed here recognize the importance of model explainability over and above model performance to an objective benchmark (i.e., a performance metric), as discussed in Sections 4.2.7 and 4.2.8. A model that has objectively strong performance is not sufficient if its strong performance cannot be explained. Our findings suggest data scientists want explanations that provide insights into the features that the model is relying on to inform its decision making. These insights allow data scientists to distinguish a high performing model that is informed by artifacts in the training data from a similarly accurate model that is informed by operationally relevant cues in the data. Using the same example described previously, one of the participants repeatedly looked at the results of an image classification model to eliminate problems in how images were classified due to nuisance features. The real-world data used in this project contained features that, in some iterations of the model, led to highly accurate classification that lacked meaning (e.g., images were classified due to size). For this data scientist, incorporating an explanation of *why* the model came to a specific classification helped to alter the features to improve performance.

Thus, with more detailed explanations of model decisions, data scientists will be better able to optimize their findings relative to the model's comprehensibility or explainability. These explanations should be meaningful in the sense that they can explain the features used to generate a specific result to allow for modification to the model. The end goal of such an explanation would be to allow data scientists to modify models from the creation stage to make them more explainable to end users. Developing explainable AI for end users is a challenging research effort that involves finding the right match between the model explanation and the end user's goals, context and experience level. However, before developers can even begin to develop useful explanations for end users they must first understand the models themselves.

### Developing expertise in machine learning

In this study, participants were asked how a novice (vs. an expert) might have completed the tasks associated with the machine learning model in their projects. In some cases, the participants reported that they were a novice at the time of task completion, but had since gained more knowledge. Their response therefore described how their current expertise might have changed their approach. When describing differences between novices and experts, participants noted that expertise helped to guide what hyperparameter ranges to explore. Lack of expertise or experience also led to a lack of confidence or knowledge of what hyperparameters might have led to more performance. Participants reported that having more expertise might have helped to feel more confident in their findings.

In addition, knowing other modeling techniques or neural architectures might have led them to try different approaches. Given the breadth of possible approaches to similar problems, participants reported that expertise leads to familiarity that might have led to improved performance. In general, participants described expertise as giving them knowledge of approaches, values, or techniques that they believed might have improved the model performance and also have reduced the time necessary to reach that performance. Novices (again, this often referred to the participants themselves at an earlier time) may lack knowledge of alternative approaches, and therefore, may not have achieved the best possible performance or outcomes because they failed to try a different strategy.

Participants who were novice data scientists also mentioned that they would have preferred an additional data scientist for consultation on the project. Although senior data scientists were available for some projects, in general, novices expressed a desire to have a sounding board for potential ideas or a second team member to review code for accuracy. They described a lack of certainty that their approach was correct given the variety of possible methods, values, and techniques that can be applied in data science.

All of these issues point to a potential need for additional guidance around machine learning and deep learning for less experienced data scientists. This issue points to an emerging issue in the area of data science in general: it may not be reasonable or feasible for data scientists to remain knowledgeable or informed on all of the possible ways that a specific machine learning problem could be solved. Instead, this may be an area where additional tools could be created to help guide researchers as they explore questions of neural architecture or other variations in machine learning on their projects. Again, this is an area for potential collaboration between the fields of machine learning and human factors: identifying the tools needed to help address the problem (the domain of human factors researchers) and building those tools (the domain of data scientists). Perhaps in an ideal solution, future technology will allow for a more balanced human-machine teaming approach where data scientists work with artificial intelligence collaboratively to identify the most appropriate approach to a specific problem.

### Domain knowledge

Almost all participants reported that they either were an expert in the domain of the project (i.e., they were domain scientists who later learned data science) or that they worked consistently with a domain scientist in their project. When asked the importance of domain knowledge when applying the technique, the participants generally described domain knowledge as important for verifying the accuracy of the results against expected findings or other benchmark results. Specifically, participants noted that accuracy alone was not sufficient to evaluate the results; instead, knowledge of the domain was important to ensuring that the model was accurate in ways that were important for application. For example, in a physics-based machine learning study, one participant noted that the results needed to conform to certain physical laws. A data scientist unaware of or unfamiliar with those laws might produce a highly accurate, but nonsensical, model.

Although this is not a challenge, per se, because participants frequently highlighted the importance of domain knowledge, it was frequently mentioned as being important to project success. Thus, because participants highlighted its importance, we include this result here.

## Discussion

Participants in this study described the process of working with machine learning models and the issues and challenges encountered when doing so. The results of this study revealed several areas where data scientists, as professionals, leverage their knowledge to generate meaningful results from data using ANNs. The interviews generated a description of the process by which data scientists work with machine learning models. They also identified several areas where human factors researchers and data scientists might be able to work collaboratively to improve the process of data science and machine learning.

As already mentioned, the field of data science has grown at an astounding rate. As with any emerging field, this growth has brought new challenges for data scientists. The number of new techniques, tools, and technology that data scientists have been expected to learn and use has similarly expanded. The data scientists who participated in this study helped to identify a variety of areas where data scientists working with machine learning models might require additional tools or support. Our interviews and analysis validated several thoughts around challenges and ambiguities in model development and deployment. Common and salient themes included:

- Data collection is often disjoint from the data scientist workflow and extensive preprocessing is cumbersome and requires additional validation from domain experts.
- Platform setup and use pose a significant challenge for reproducing previous results. Underutilized tools and lack of guidance around documentation lead to longer process times and a lack of assurance that one's model is performing in a consistent manner.
- Providing tools for assessing assurance that the model is performing (and will perform) as intended is high desired. Creating standards here would aid in model deployment.

In addition to identifying specific areas of need, this study also highlighted that the challenges encountered by data scientists are complex and require the inputs of a multidisciplinary community of experts. For example, addressing the issues in hyperparameter optimization will likely require additional research from data scientists, exploration and evaluation by human factors experts, and support from software engineers or other computing experts. Working with high-performance computing resources not only requires training and skills development for data scientists, it also requires knowledge of the hardware provided by computer scientists and engineers. As machine learning and deep learning increase in their sophistication and develop toward artificial intelligence-like capabilities, continuing to build collaboration amongst these fields is increasingly important. We acknowledge that many of these issues are known to data scientists; however, this study represents an empirical demonstration of those issues with a sample of data scientists working in the field. This study also presents an overview of the most common challenges in deep learning; even if these issues are known to data scientists, this study identified and reviewed those challenges in a systematic manner. In addition, the results here help to describe the ways that human factors methods might be deployed to help address the common issues in deep learning.

It is important to acknowledge that this study focused primarily on data scientists working with ANNs in applied research context. Other modeling approaches may use a different process or encounter different challenges. For instance, due to the expertise of the data scientist or their specific domain application, the use of an ANN as a modeling approach was assumed. Other data scientists may have an explicit process step devoted to selecting a modeling approach, and again, once selected, may use a different process than the one presented here. In addition, some of the challenges presented (e.g., explainability) do not apply to other types of data science approaches (e.g., linear regression) or even machine learning approaches. There is also some potential that the findings might not be reflective of broader data scientists' approaches because all of the researchers sampled here worked at the same organization, and certainly institutional norms develop. We also acknowledge that the data scientists here are relatively new to the field, with around 3 or 4 years of experience, and different issues might emerge with a sample of more experienced data scientists. Finally, many of the projects focused on the use of machine learning models within applied research rather than more exploratory or basic research. The process or challenges of working with machine learning models might differ in those different contexts. Nonetheless, the findings do provide some insight into the unique process and challenges used by data scientists working with ANNs.

We do not suggest that human factors expertise is a panacea for the issues and challenges confronting data scientists who work with machine learning models. Instead, we suggest that, as the machine learning develops, human factors experts might be able to contribute by helping to guide tool development as well as apply theories, and knowledge developed over decades of research in human factors and related fields (e.g., human-computer interaction). The recommendations and areas for collaboration described in this report are some ways that future work could help to provide better tools, knowledge, and guidance to machine learning scientists.

## Data availability statement

The raw data supporting the conclusions of this article will be made available by the authors, without undue reservation.

## Ethics statement

The studies involving human participants were reviewed and approved by Pacific Northwest National Laboratory. The patients/participants provided their written informed consent to participate in this study.

## Author contributions

CF and BJ conceived of the presented idea. CF developed the interview method and assisted in the interviews. JB conducted the interviews and the analysis and took the lead in writing the manuscript with input from all authors. All authors contributed to the article and approved the submitted version.

## Footnotes

^1^We recognize that having a single reviewer and coder conduct the analysis is not ideal, and we acknowledge this as a limitation of this study.

^2^Although the focus of this effort was intended to be ANNs, and participants generally specialized in work involving ANNs, a single participant expressed difficulties in identifying a suitable project involving an ANN to discuss. As a result, the interview was conducted using another machine learning project on which the participant worked. However, because many of the process steps were similar, we choose to present those results here; the findings do not significantly change if this participant's responses are excluded.
