# Corrigendum: Opportunities for human factors in machine learning

**DOI:** 10.3389/frai.2023.1327954

**Published:** 2023-11-17

**Authors:** Jessica A. Baweja, Corey K. Fallon, Brett A. Jefferson

**Affiliations:** Pacific Northwest National Laboratory, Richland, WA, United States

**Keywords:** human factors, machine learning, neural networks, data science, artificial intelligence

In the published article, there was an error where the Acknowledgments statement was not included. The missing Acknowledgments statement appears below.

## Acknowledgments

The research described in this paper is part of the MARS Initiative at Pacific Northwest National Laboratory. It was conducted under the Laboratory Directed Research and Development Program at PNNL, a multiprogram national laboratory operated by Battelle for the U.S. Department of Energy.

The authors apologize for this error and state that this does not change the scientific conclusions of the article in any way. The original article has been updated.

